# Ag-Doped ZnO Quantum Dots for Fluorescence-Based Detection of Trace Cr(VI) from River Water and Fish samples

**DOI:** 10.1007/s10895-026-04750-3

**Published:** 2026-04-16

**Authors:** Genet Aregay Shifera, Shimeles Addisu Kitte, Tamene Tadesse Beyene, Abera Gure

**Affiliations:** College of Natural Sciences, Department of Chemistry, Jimma University, P. O. Box 378, Jimma, Ethiopia

**Keywords:** Ag-doped ZnO, Cr(VI), Fluorescence detection, Quantum dots, Water and Fish samples

## Abstract

The co-precipitation process was used to create silver-doped zinc oxide. Fluorescence spectrophotometry, XRD, FT-IR, BET, TGA, and UV-Vis were used to characterize it. When stimulated at 360 nm, the Ag/ZnO QDs displayed intense fluorescence emission at 508 nm and were highly selective for Cr(VI). A calibration curve was plotted for different concentration levels of Cr(VI) ranging from 0 to 12 mg/L, with an R² value of 0.998 and the LOD and LOQ at 3.2 µg/L and 11 µg/L, respectively. Three concentration levels, 0.8, 5.6, and 10.4 mg/L, were used to examine the precision of the devised approach; relative standard deviation values did not surpass 5.4%, showing good reproducibility. By adding known quantities of Cr(VI) to the water and fish samples, the method’s recovery was assessed; the results ranged from 77.5% to 112.5%. Ultimately, the suggested approach demonstrated outstanding sensitivity, selectivity, and repeatability for the detection of Cr(VI) in fish and water samples.

**Graphical Abstract:**

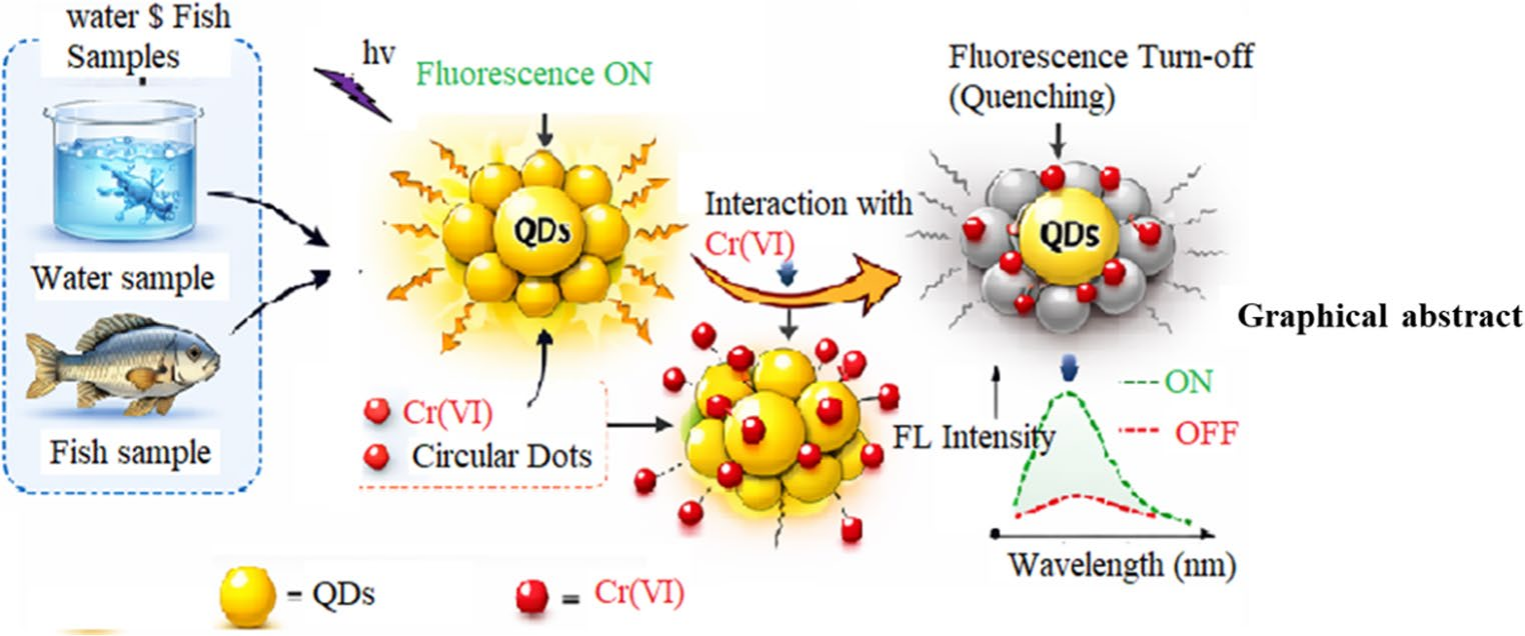

## Introduction

Chromium in food and water is a serious environmental and public health issue in the twenty-first century, which is a result of industrialization and population growth [1]; common industrial uses of chromium include metal smelting, dyeing, and tanning leather. These processes release massive amounts of chromium into the environment, contaminating food and drinking water. The environmental sample of chromium contains two of its best stable oxidation states, i.e., the Cr(III) and the Cr(VI). Cr(III) is necessary and not harmful to mammals [1]; Cr(VI) is of special worry due to its toxicity and carcinogenicity, which pose significant hazards to the environment and human health [2]. After release, it can spread by leaching, volatilization, and surface runoff, causing widespread aquatic pollution [3]; Even at trace levels, Cr(VI) can cause liver and kidney damage, in addition to increasing the risk of cancer in humans [4]; therefore, the development of rapid, simple, highly sensitive, and consistent methods for Cr(VI) detection is essential for effective watching and pollution control.

Several analytical techniques have been utilized to detect Cr(VI), such as inductively coupled plasma mass spectrometry (ICP-MS) [6], atomic absorption spectroscopy (AAS) [5], inductively coupled plasma atomic emission spectrometry (ICP-AES) [7], UV–Vis spectrophotometry [4, 8], colorimetric methods [9, 10], and X-ray fluorescence spectrophotometry [11], which have high sensitivity and precision but are usually expensive, labor intensive, and not applicable for on-site or real-time monitoring. In contrast, fluorescence-based approaches have been increasingly popular due to their high selectivity, sensitivity, rapid response, minimal sample preparation, wide linear dynamic range, and operational simplicity [12–15]; the incorporation of nanomaterials further enhances the performance of fluorescent sensors by increasing surface area for Cr(VI) interaction and minimizing response times [16].

Nanomaterials have been thoroughly studied as adsorbents, sensors, and catalysts because of their high reactivity and large surface area [17]. Fluorescent nanomaterials such as quantum dots, carbon dots, and other zero-dimensional nanostructures (e.g., CdS, CdSe, CdTe, and PbS) are particularly attractive for metal ion detection because of their broad absorption spectra, high Stokes shifts, tunable emission, low cost, and facile synthesis [7, 9, 12, 18]. Among these, metal oxide semiconductor QDs, especially zinc oxide (ZnO) QDs, which are notable for their wide band gap, low toxicity, photostability, high quantum yield, and strong luminescence [19]. Pure ZnO QDs often exhibit low fluorescence intensity, which results in poor detection efficiency despite their other beneficial properties.

ZnO modification with noble metals or organic molecules has been investigated as a way to get around these restrictions, greatly enhancing their conductivity, stability, and sensing capabilities [16]; examples include functionalized ZnO QDs for the selective detection of Cr(III), Cu(II), Cr(VI), and Hg(II) ions and Ag-ZnO/polyaniline nanocomposite for pesticide detection [19–25]. Then, silver-doped ZnO (Ag/ZnO) QDs have demonstrated superior optical properties, strong fluorescence emission, and excellent photostability, making them promising candidates for pollutant sensing [25]. The quenching of fluorescence intensity induced by Cr(VI) interaction with Ag/ZnO QDs offers a sensitive and selective detection mechanism.

Several QDs have been described for Cr(VI) [16, 26–28], including graphitic carbon nitride hybrids, carbon quantum dots, Fe₃O₄ composites, ZnO/urea, and glutathione-capped CdTe. Research on the use of Ag/ZnO QDs is not yet underway. This work advises a new fluorescence-based technique using Ag/ZnO QDs for the quick and precise detection of trace Cr(VI) in fish and water matrices. Besides its high possibilities for public health defence and environmental monitoring, this method is basic, inexpensive, and very dependable.

## Materials and Methods

### Chemicals and Reagents

Analytical grade of all reagents and chemicals were employed. Potassium dihydrogen phosphate (KH₂PO₄), and dipotassium hydrogen phosphate (K₂HPO₄) were obtained from ICE Chemical Reagent (India). Potassium dichromate (K₂Cr₂O₇), and nitric acid (HNO₃) were obtained from BDH Chemicals Ltd (Poole, England). Lead nitrate (Pb(NO₃)₂), cobalt nitrate (Co(NO₃)₂), cadmium nitrate (Cd(NO₃)₂), zinc nitrate (Zn(NO₃)₂). 6H_2_O, and chromium nitrate (Cr(NO₃)₃) were purchased from Finekem Pvt. Ltd (India). Sodium chloride (NaCl) and sodium carbonate (Na₂CO₃) were supplied by Fisher Scientific (UK). Sodium acetate (CH₃COONa) was obtained from Merck (Darmstadt, Germany) and silver nitrate (AgNO₃) was obtained from Kiran Light Laboratory (India).

A stock solution of Cr(VI), 1000 mg/L, was prepared by dissolving 2.83 g of K₂Cr₂O₄ in 0.01 mol/L HNO₃. The stock solution was then diluted with ultrapure water to create the working solutions each day. Additionally, KH₂PO₄ and K₂HPO₄ were used to prepare a phosphate buffer.

## Instrument and Apparatus

A fluorescence spectrophotometer (DW-S97, Drawell, Shanghai, China) was employed to quantitatively analyze Cr(VI) and to study the optical properties of the nanomaterials. A portable pH meter made by Hanna Instruments in Póvoa de Varzim, Portugal, was used to determine the sample solution’s pH. The Rigaku MiniFlex 300 or 600 diffractometer for X-ray diffraction (XRD). The company Rigaku, which makes instruments, is based in Japan. The Brunauer-Emmett-Teller (BET) method (Nova Station B, Micromeritics Instrument Corporation, United States) was used to measure the surface area of solid materials, and Fourier-transform infrared spectroscopy (FT-IR, JASCO, Japan) was used to identify the functional groups of the synthesized materials. The thermal stability of the synthesized materials was being examined using thermogravimetric analysis. To study the optical properties of Ag-doped ZnO and ZnO, UV–Vis spectrophotometry (double beam, SPECORD 200 PLUS, Analytik Jena, Germany) was utilized. A centrifuge (Jiangsu Zhenji Instruments Co., Model 800, Ltd., Jiangsu, China) and Falcon centrifuge tubes with a capacity of 15 mL were also utilized in the process of preparing the samples.

## Sample Collection

Water samples were taken from the Gilgel Gibe-I dam in the Jimma zone of the Oromia region of Ethiopia and from the rivers Nedi, Yedi, and Guda, which are its tributaries. The University of Jimma Aquaculture and Fisheries Laboratory has provided samples of African catfish (Clarias gariepinus).

## ZnO Synthesis

The co-precipitation technique was used to create ZnO nanoparticles. First, deionized water was utilized (80 mL) to dissolve 3 g of Zn(NO₃)₂·6 H₂O, which was then stirred for 1 h at 80 °C. To create a clear basic solution, 20 mL of deionized water was added to another beaker along with 6 g of NaOH, and the mixture was stirred for 30 min. The zinc nitrate solution was then stirred continuously for 2 h while the NaOH solution was added dropwise. After 16 h at room temperature, a white precipitate formed. Following four rounds of washing with deionized water to get rid of any leftover reactants and byproducts, the product was then further cleaned with ethanol to get rid of any last contaminants; the purified ZnO was filtered and dried at 80 °C in an oven for 24 h. After the product was calcined for 2 h at 550 °C in a muffle furnace, the resulting ZnO nanoparticles were ground with a mortar and pestle into a fine powder [29].

## Ag-doped ZnO Synthesis

The co-precipitation technique was used to create Ag-doped ZnO nanoparticles. Zinc nitrate (2.97 g) and silver nitrate (0.03 g) were dissolved in 70 mL and 10 mL of distilled water, respectively. After 30 min of separate stirring, two solutions were combined and stirred for an additional 30 min at 80 °C. Deionized water (20 mL) was used to dissolve 6 g of NaOH, and the liquid was agitated for 30 min. Ag-doped ZnO was co-precipitated after this solution was added dropwise to the mixed nitrate solution while being constantly stirred at 80 °C for 1 h; a black precipitate was created after the mixture was permitted to sit at 25 °C for 16 h; the precipitate was dried in an oven at 80 °C for 24 h, calcined at 550 °C for 2 h in a muffle furnace, and rinsed four times with deionized water and ethanol to eliminate any materials that haven’t yet reacted. Ultimately, the material was ground into a fine powder using a mortar and pestle [29].

### Characterization

The optical characteristics of the as-synthesized nanostructures were examined using UV-visible spectrophotometry (double beam, SPECORD 200 PLUS, Analytik Jena, Germany) and fluorescence spectrophotometry (DW-S97, Drawell, Shanghai, China). The functional groups and surface structures of the samples were determined using Fourier transform infrared (FT-IR) spectroscopy (JASCO, Japan) in the 4000–400 cm⁻¹ range using KBr plates. TGA analysis was used to describe thermal stability in the temperature range of 0 to 900 °C. The Brunauer-Emmett-Teller (BET) technique was used to determine the materials’ surface area (Nova Station B, Micromeritics Instrument Corporation, United States).

## Procedure for Cr(VI) Determination

The Ag-doped ZnO probe was prepared by adding 2.5 mL of Ag-doped ZnO solution (1 mg/mL) to a 10.0 mL volumetric flask and then adding deionized water to dilute the mixture to volume. Subsequently, small amounts of various Cr(VI) solutions were added until the final concentrations ranged from 0 to 12 mg/L. Then the pH was adjusted to 6, and the reaction time was set for 25 min, resulting in maximum and stable quenching. The Ag-doped ZnO emission at 508 nm was extinguished, displaying a “turn-off” fluorescence response. The same procedure was used to detect other metal and non-metal ions (such as Cd²⁺, Co²⁺, Zn²⁺, Fe²⁺, Pb²⁺, Ni²⁺, Cr³⁺, CH₃COO⁻, CO₃²⁻, PO₄³⁻, SO₄²⁻, and Cl⁻) to assess the impact of coexisting ions on the Ag/ZnO QDs for the selectivity of the target analyte.

## Pre-treatments of Water and Fish Sample

Water samples from the Gilgel Gibe-I Dam’s three tributary rivers, the Nedi, Yedi, and Nada Guda, were collected for examination and detection. After removing any suspended solids from the water samples using Whatman filter paper, they were kept at 4 °C until they were required once more. Samples of catfish were put to sleep and dried at 105 °C in an oven until their weight remained constant. A mortar and pestle were used to grind the dried samples into a fine powder. 5 g of the homogenized catfish dust was weighed, mixed with 90 mL of water (deionized), and then ultrasonically stirred for 30 min. After 15 min of centrifugation to eliminate any remaining residues, the supernatant was collected and diluted with 0.1 M phosphate buffer (pH 6.0). The prepared sample was spiked with standard Cr(VI) solutions at concentrations of 0.8, 5.6, and 10.4 mg/L. Fluorescence spectrophotometry was utilized to analyze each concentration in triplicate [8, 30].

## Results and Discussion

### Characterization of QDs

#### XRD Analysis

**Fig. 1:**
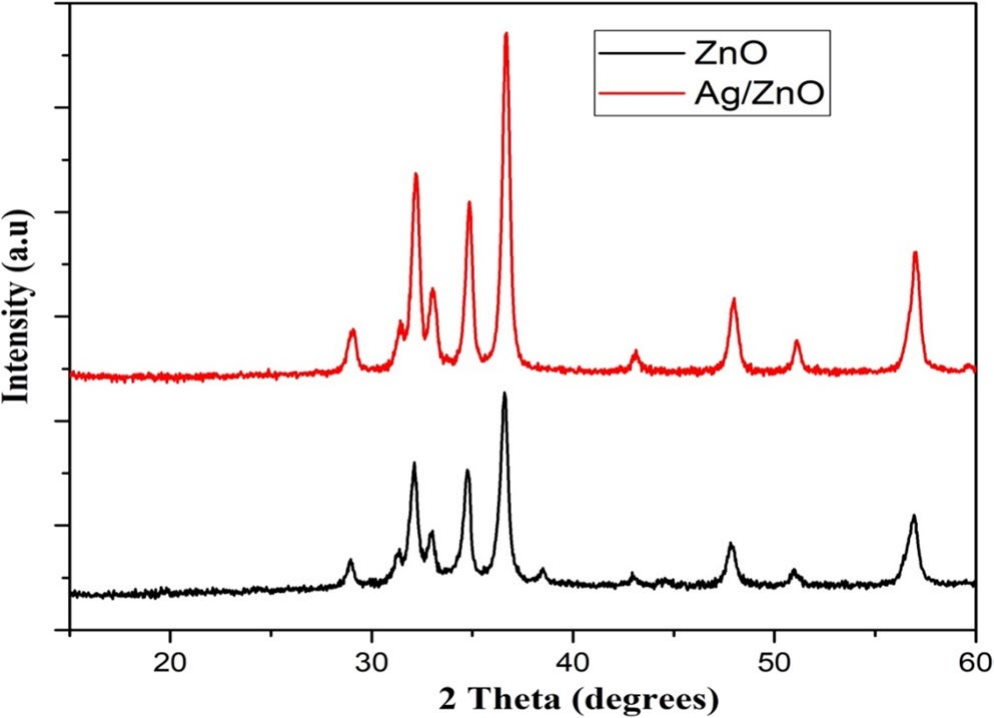
Ag-doped and un-doped ZnO nanoparticles’ XRD patterns

To assess the crystal size and property of these materials, Fig. 1 displayed the X-ray diffraction peak of the Ag-doped ZnO and ZnO nanoparticles. The planes confirm the exceptional purity of the powder and support the hexagonal wurtzite structure of ZnO. In addition, when Ag ions are substituted with ZnO, the diffraction intensity peaks as the Ag content increases. The average size of the ZnO and Ag-doped ZnO nanoparticles was confirmed using Scherrer’s formula based on the noticeable peak in the XRD [31].

$$\mathrm{D}=\frac{0.9\lambda}{\beta\:\cos\:\theta}$$

Where λ is the wavelength of the X-ray, β is the full width at half maximum (FWHM), and θ is the Bragg diffraction angle of the XRD peak. It was calculated that the average sizes of Ag-doped ZnO and ZnO nanoparticles were 13.7 and 10.2 nm, respectively.

### BET Surface Area

The Brunauer-Emmett-Teller (BET) method uses the adsorption of gas to determine the surface area of solid materials. The BET surface area of Ag-doped ZnO (192.733 m²/g) is much larger than that of pure ZnO (113.245 m²/g). This finding shows that the silver NPs were successfully loaded onto the surface of ZnO, which increased the surface area by creating more active sites and changing the shape of the material. More surface area typically results in more effective interactions with reactants, higher adsorption capacity, and improved detection efficiency [31]. Table 1 shows the surface area of as-synthesized materials compared with previously reported materials.

**Table 1:** The surface area obtained in this study was compared with previously reported values

Surface area (m²/g)		
ZnO	Ag doped ZnO	Ref
34	150	[31]
5.7	5.8	[32]
34.48	38.06	[33]
18	31	[34]
113.245	192.733	This study

### FT-IR Analysis

**Fig. 2:**
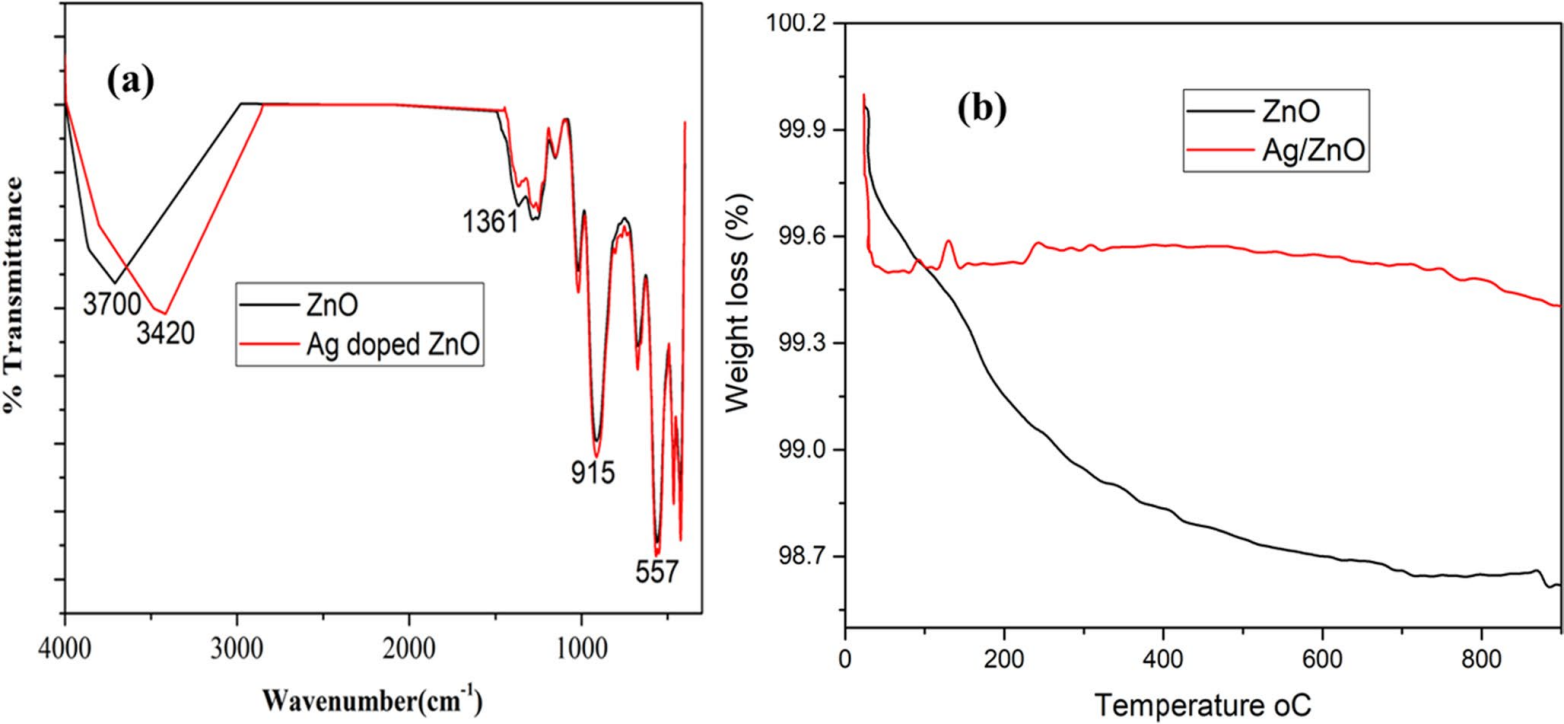
The ZnO and Ag-ZnO doped nanoparticles’ FT-IR and TGA spectra are shown in (**a**) and (**b**) respectively

ZnO and Ag–ZnO FT-IR spectra recorded between 400 and 4000 cm⁻¹ were displayed in Fig. 2(a). In the ZnO spectrum, the stretching vibrations of the -OH group from adsorbed water is responsible for a broad absorption band at 3700 cm⁻¹ [35]. The peak at 915 cm⁻¹ and 1361 cm⁻¹ are also associated with the –OH group bending vibrations. The strong absorption peak at 557 cm⁻¹ is caused by Zn–O stretching vibrations [36]. Ag-doped ZnO was found to exhibit a shift in the hydroxyl band to 3420 cm⁻¹, suggesting that surface hydroxyl groups are crucial for the sensing activity [16].

### TGA Analysis

As seen in Fig. 2(b), a thermogravimetric analysis was utilized to assess the thermal stability of the materials as synthesized. The Ag-doped nanomaterials lost 0.43% and 1.25% of their total weight between 0 and 900 °C, respectively. The vaporization of chemically and physically adsorbed water on the material’s surface is the cause of the initial mass loss seen in both samples at 500 °C [37, 38]. Both ZnO and the Ag-doped ZnO showed almost constant weight loss at temperatures above 500 °C, indicating that the synthesized samples were more thermally stable.

### Ag/ZnO QDs Optical Property

**Fig. 3:**
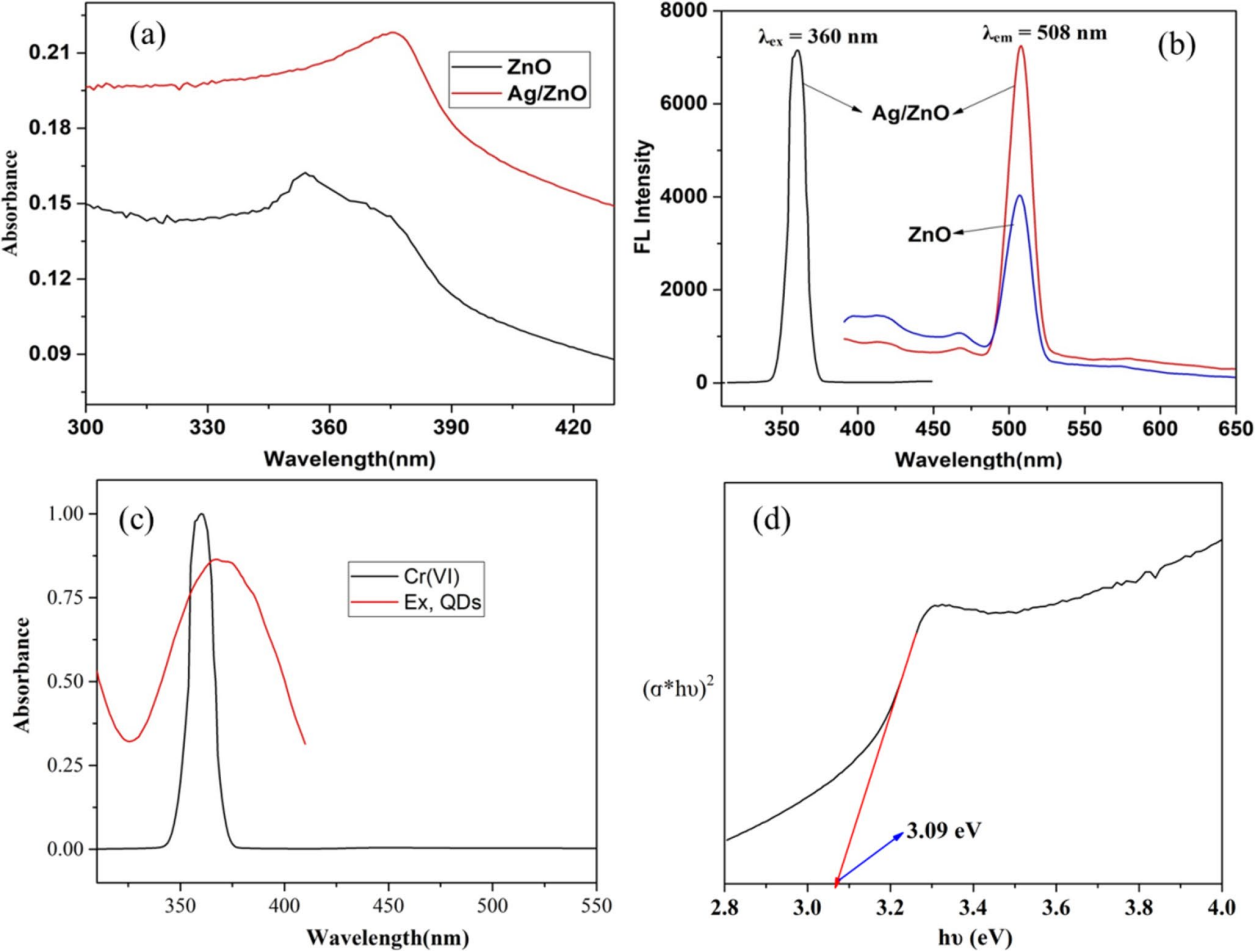
(**a**) and (**c**) QD and Cr(VI) UV-Vis spectra, (**b**) Ag-ZnO and pure ZnO QD fluorescence spectra, and (**d**) The Energy band gap of Ag-ZnO

Figure 3(a) shows the UV-Vis absorption spectra of undoped and Ag-doped ZnO nanoparticles. The Ag-doped ZnO showed a slight red shift with the absorption edge appearing at 375 nm, whereas the undoped ZnO showed a maximum absorption at 353 nm. The addition of Ag to the ZnO matrix is responsible for this shift toward a higher wavelength, which shows a reduction in the optical band gap [39].

Ag-doped ZnO improved optical qualities and substantial chemical sensing capabilities make it a promising material for identifying different pollutants in environmental and related applications. The Ag-ZnO QDs have an emission wavelength of 508 nm and an excitation wavelength of 360 nm, as shown in Fig. 3(b). Moreover, the Ag nanoparticles significantly increase the fluorescence intensity of pure ZnO QDs by acting as plasmonic antennas to increase ZnO excitation and produce stronger UV emission. When Ag is present in core-shell configurations or as a surface coating, this effect is frequently seen [40].

### Parameters Optimization

**Fig. 4:**
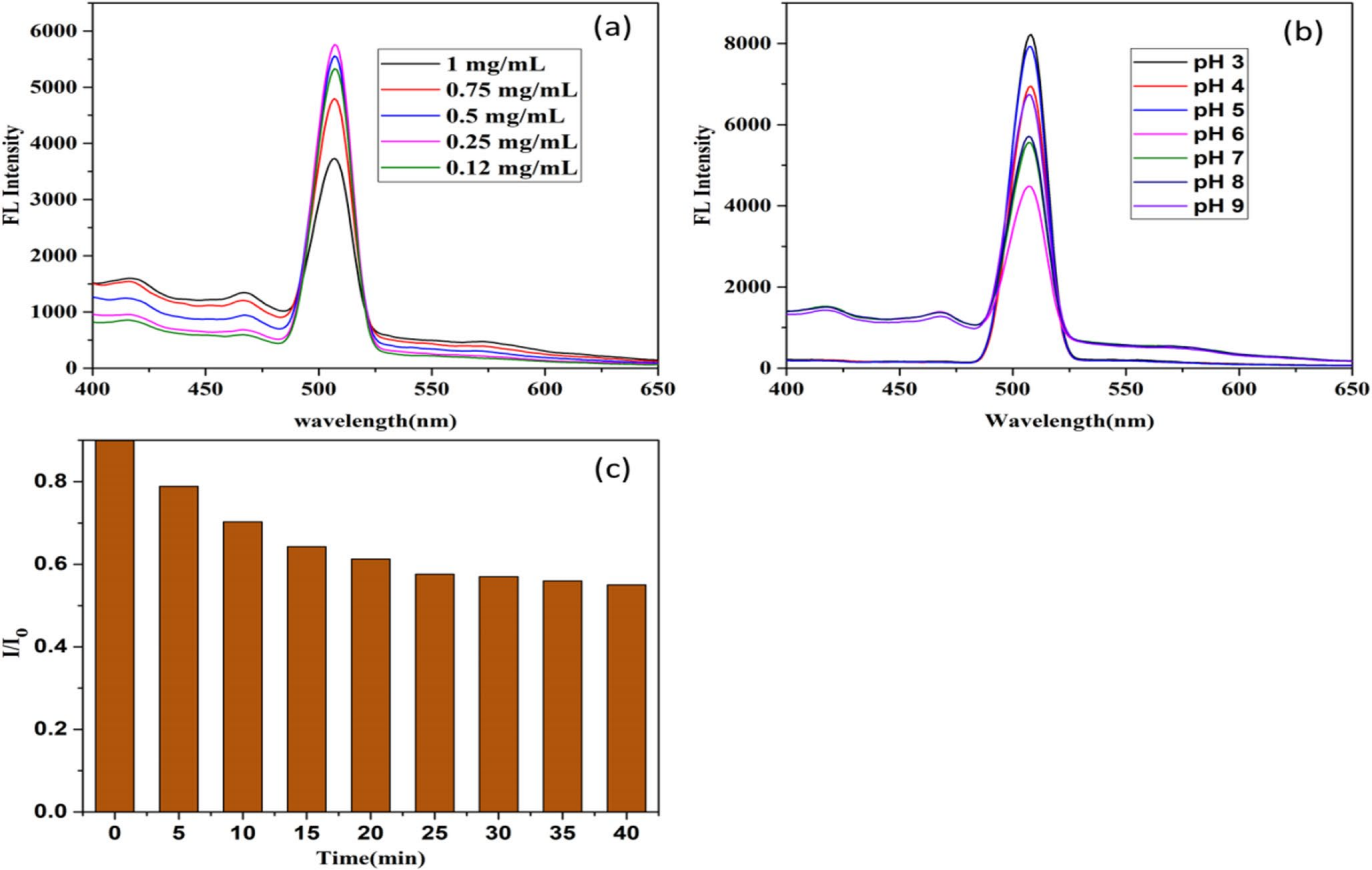
Impact of (**a**) Ag-ZnO concentration, (**b**) pH of solution, and (**c**) duration

The concentration of Ag/ZnO QDs, solution pH, and reaction time between Ag/ZnO QDs and Cr(VI) were systematically optimized to enhance detection sensitivity. The quenching efficiency was evaluated using the ratio I/I₀, where I₀ and I represent the fluorescence intensities in the absence and presence of Cr(VI). Maximum detection sensitivity was obtained at pH 6.0, an Ag/ZnO QD concentration in the 0.25 mg mL⁻¹, and a reaction time of 25 min, as shown in Fig. 4(a–c).

### Quenching Mechanism

When Cr(VI) is present, the fluorescent probe must be put out using one of two techniques: (1) static extinguishment, in which the probe and Cr(VI) combine to form a non-fluorescent complex; or (2) dynamic extinguishment, in which Cr(VI) collides with the probe to put it out. (3) When the probe is excited or Cr(VI) absorbs the emission light, the inner filter effect (IFE) takes place; (4) Photoinduced electron transfer (PET): when the excited probe absorbs electrons from the probe, the fluorescence is eliminated [41].

Using Ag-doped ZnO as a fluorescent probe, a novel spectrofluorimetric technique was created for the straightforward and selective measurement of Cr(VI). Cr(VI) significantly reduced the fluorescence intensity at 508 nm under the ideal conditions mentioned; the Cr(VI) aqueous solution’s UV–Vis absorption displayed a single peak at 367 nm (Fig. 3c). The maximum excitation wavelength was 360 nm, and Ag-doped ZnO showed the strongest emission bands of fluorescence at 508 nm. The overlap between the UV-Vis absorption peak of Cr(VI) and the fluorescence excitation peak of Ag-doped ZnO suggests that if Cr(VI) absorbs the excitation wavelength, the fluorescence emission of Ag-doped ZnO would be extinguished by either the photoinduced electron transfer or inner filter effect [42]. The IFE is not an actual quenching effect involving direct molecular interaction; it is an optical artifact caused by either the excited or emitted light being absorbed by the quencher, which acts as a filter to lessen the quantity of light that reaches the fluorophore or detector. In contrast, in PET, an electron is transferred directly from the donor to the quencher molecule, or vice versa; this transfer competes with the fluorescence [41].

As shown in Fig. 3(d), the direct electronic band gap (Eg) of Ag/ZnO is 3.09 eV. To determine the photoreaction mechanism of Ag/ZnO, the potentials of the conduction band (CB) and valance band (VB) were calculated using Eqs. 1 and 2:

1 $$\mathrm{E}_\mathrm{VB}=\mathrm{E}_\mathrm{CB}+\mathrm{E}_\mathrm{g}$$

2 $$\mathrm{E}_\mathrm{CB}=\mathrm{X}-\mathrm{Ee}-\mathrm{Eg}$$

Where E_VB_ is the potential of VB, E_CB_ is the potential of CB, Eg is the band gap of Ag/ZnO (3.09 eV), χ is the absolute electronegativity of ZnO (5.89 eV), and Ee is the constant relative to the standard hydrogen electrode (4.5 eV).

**Fig. 5:**
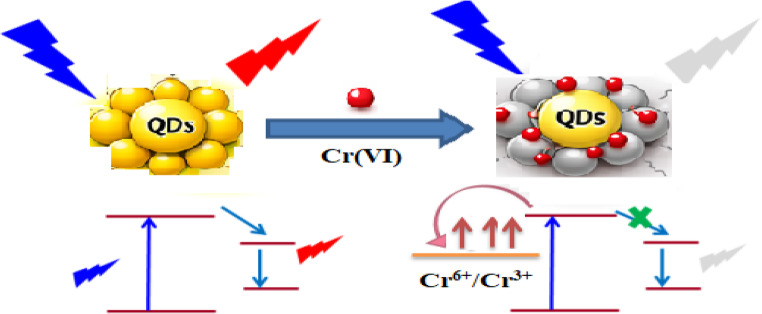
Schematic representation of the Ag/ZnO-based fluorescence probe used to measure Cr(VI) concentration

The calculated E_CB_ and E_VB_ were found to be -1.7 and 1.39 eV, respectively. The redox potential of Cr⁶⁺/Cr³⁺ is 1.33 eV, which lies between the CB and VB of Ag/ZnO [51]. Thus, the photoinduced electron in the CB of Ag/ZnO can transfer to the unfilled d-orbital of Cr (VI), resulting in fluorescence quenching via photoinduced electron transfer (PET). As shown in Fig. 5, the electron transfer from the excited Ag-doped ZnO to Cr(VI) is a PET process; therefore, the proposed quenching mechanism is PET.

### Selectivity and Sensitivity of the Probe

**Fig. 6:**
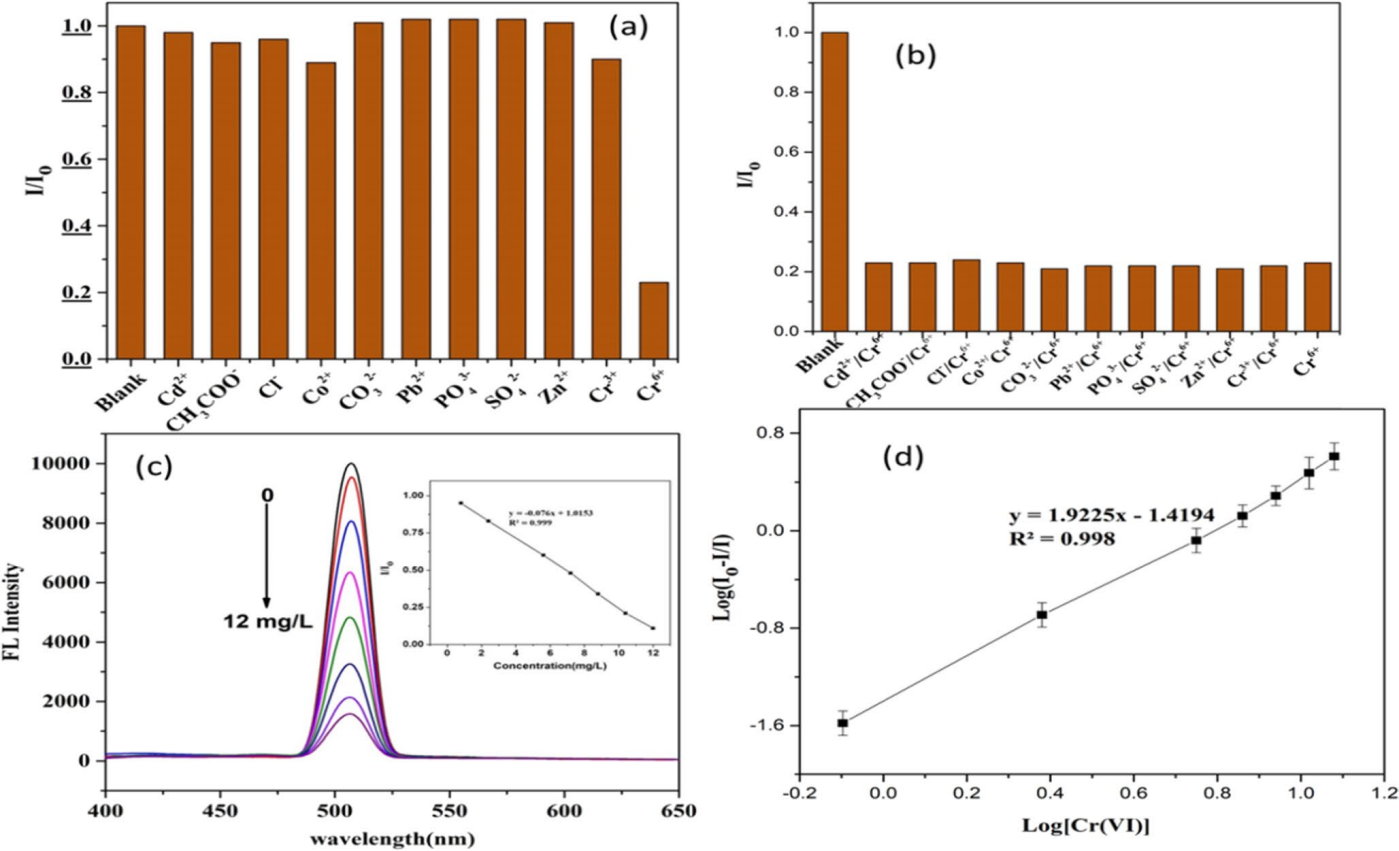
(**a**) and (**b**) the method’s selectivity, and (**c**) and (**d**) the Cr(VI) calibration curve

Fluorescence intensity was evaluated to investigate the selectivity of Ag/ZnO QDs for Cr(VI) in the presence of a fixed concentration (12 mg/L) of several potentially interfering metal and non-metal ions, including Cd²⁺, Pb²⁺, Zn²⁺, Co²⁺, Cr³⁺, CH₃COO⁻, CO₃²⁻, PO₄³⁻, and SO₄²⁻. Selectivity is a crucial parameter in determining the dependability of a fluorescent sensor. After a 25-min sonication period, the experiments were conducted at pH 6. When compared to other tested ions, Figs. 6(a) and (b) demonstrate the remarkable selectivity of Ag/ZnO QDs toward Cr(VI).

Figure 6(c) displays the fluorescence spectra of Ag/ZnO QDs after various quantities of Cr(VI) were added. As the concentration of Cr(VI) rises from 0 to 12 mg/L, the fluorescence intensity steadily declines. The calibration equation, I/I₀ = −0.076 × [Cr(VI)] (mg/L) + 1.015, shows a strong linear relationship with a high correlation coefficient of R² = 0.999.

### Method Validation

The calibration curve generated with seven Cr(VI) concentrations from 0 to 12 mg/L is displayed in Fig. 6(d); each concentration was analyzed twice, and the fluorescence response was recorded three times to ensure data consistency and accuracy. The logarithms of the fluorescence response and the Cr(VI) concentration were compared to plot the curve. The analysis resulted in a linear equation, y = 1.9255x − 1.4194, with an excellent correlation coefficient of R² = 0.998. As low as 0.062 µM (0.0032 mg/L), the detection limit of the Ag/ZnO quantum dot showed a high level of sensitivity.

#### Precision Studies

Precision of the approach was appraised using three levels of Cr(VI) concentration (0.8, 5.6, and 10.4 mg/L), with a focus on both repeatability (intra-day precision) and inter-day precision (within-laboratory reproducibility). Intra-day precision was assessed by measuring the fluorescence response three times for each detection and evaluating every concentration level twice a day, in the morning and the afternoon. Over the course of three days, two samples per concentration level were analyzed daily in duplicate to evaluate inter-day precision. Strong reproducibility of the process was indicated by inter- and intra-day measurements with RSDs of ≤ 5.4%.

### Analysis of Real Samples and recovery studies

The applicability of the technique was assessed using three water samples (Yedi, Nedi, and Nada Guda) and fish samples from the Gilgel Gibe-I Hydroelectric Dam reservoir and tributary rivers (Nedi, Yedi, and Nada Guda). Cr(VI) was detected in Yedi and Nedi water samples with concentrations of 0.27 ± 0.5 mg/L and 0.45 ± 0.3 mg/L, respectively; however, it was not detected in the Nada Guda water and fish samples.

To perform percent recovery (%R) studies, water and fish matrices were spiked at three concentration levels (corresponding to the three levels used for the precision evaluation) and prepared in duplicate; each detection was performed in triplicate. The recovery values (%R ± RSD) ranged from 77.5 ± 2.4 to 112.5 ± 0.4, which are all within an acceptable range, and therefore the method is accurate and reliable for real sample analysis (Table 2).

**Table 2:** The method’s recovery

Conc. spiked (mg/L)		%*R* ± RSD		
	Yedi	Nedi	Nada guda	Fish
0.8	105.0 ± 1.6	93.7 ± 0.7	90.0 ± 2.2	77.5 ± 2.4
5.6	106.5 ± 3.1	106.3 ± 1.2	93.9 ± 1.9	88.2 ± 1.1
10.4	111.9 ± 2.7	108.7 ± 2.3	100.4 ± 3.7	112.5 ± 0.4

#### Comparing the Approach to other Documented Techniques

A suggested technique was likened to earlier documented approaches for Cr(VI) determination in various matrices, including water. Contrasted to the existing methods, the Ag-ZnO QD-based approach has a broader linear range and a lesser detection limit, as indicated in Table 3, underscoring its increased efficacy and sensitivity for Cr(VI) detection.

**Table 3:** Comparing the current method for detecting Cr(VI) with earlier reports

Fluorescent probe	Linear range	limit of detection	Ref.
N, S-CDs	1–40 µM /0.05-2 mg/L	0.058 µM/0.0030 mg/L	[43]
N-GQDs	0.1–100 µM/0.005-5 mg/L	0.091 µM/0.0047 mg/L	[44]
CD	0.05-10.0 µM/0.003–0.52 mg/L	0.015 µM/0.0008 mg/L	[45]
CQD	0.01–50 µM /0.0005-2.6 mg/L	0.52µM/0.027 mg/L	[46]
PANI/Ag/GO QDs	0.2–144 µM/0.01–7.5 mg/L	0.12 µM/0.006 mg/L	[47]
N, P-CDs	0–87 µM/0-4.5 mg/L	0.18 µM/0.009 mg/L	[48]
(Cu-CDs	0–80 µM/0 -4.2 mg/L	0.186 µM/0.0097 mg/L	[49]
Zr-N-CDs	0.4–100 µM/0.02–5 mg/L	0.52 µM/0.027 mg/L	[50]
Ag-ZnO	0 -230 µM/0–12 mg/L	0.062 µM/0.0032 mg/L	This study

## Conclusion

Ag/ZnO QDs were effectively employed to propose a fluorescence-based detection of Cr(VI). To make the Ag/ZnO QDs, silver and zinc nitrates were utilized as precursors in the co-precipitation process. To examine the crystalline property, surface functionality, surface area, optical properties, and thermal stability of the resultant QDs, XRD, FT-IR, BET, UV-Vis, fluorescence spectroscopy (FS), and TGA were used. At 508 nm, the QDs displayed a strong fluorescence emission when excited at 360 nm. In the occurrence of potentially coexisting ions such as Cd²⁺, Co²⁺, Zn²⁺, Fe²⁺, Pb²⁺, Ni²⁺, Cr³⁺, CH₃COO⁻, CO₃²⁻, PO₄³⁻, SO₄²⁻, and Cl⁻, Ag/ZnO QDs generally demonstrated high sensitivity and selectivity toward Cr(VI). The fluorescence probe displayed a linear response to Cr(VI) concentrations ranging from 0 to 12 mg/L. These findings imply that the Ag/ZnO QD may be a quick, accurate, and highly sensitive way to identify Cr(VI) in actual samples.

## Data Availability

No datasets were generated or analyzed during the current study; therefore, data sharing is not applicable.

## References

[1] Cai F, Liu X, Liu S, Liu H, Huang Y (2014) A Simple One-Pot Synthesis of Highly Fl Uorescent Nitrogen-Doped Graphene Quantum Dots for the Detection of Cr (VI) in Aqueous Media. RSC Adv 4:52016–52022. 10.1039/c4ra09320h

[2] Huang G, Ye H, Mo X, Hao R, Huang G, Liang J, Wang D, Xiao X, Zhu W (2024) Coating Fe_3_O_4_ Quantum Dots with Glutamic Acid to Achieve Enhanced Catalysis for Facile and Sensitive Detection of Chromium(VI) in Water. Anal Methods 16:7161–7178. 10.1039/d4ay01408a 39297415 10.1039/d4ay01408a

[3] Kochito J, Gure A, Abdisa N, Beyene TT, Femi OE (2024) Magnetic Biochar Nanocomposites of Coffee Husk and Khat (Catha Edulis) Leftover for Removal of Cr (VI) from Wastewater. Curr Opin Green Sustain Chem 8:100403. 10.1016/j.crgsc.2024.100403 10.1155/2024/7585145 PMC10907103 38434937

[4] Kebede A, Tilachew G, Chirfa G, Gure A (2022) Effervescence Assisted Dispersive Liquid-Liquid Microextraction for Spectrophotometric Determination of Chromium (VI) in Water, Tannery Effluent, Milk, and Orange Juice Samples. S Afr J Chem 76:127–133. https://doi.org/https://journals.co.za/content/journal/chem/

[5] Panichev N, Mandiwana K, Kataeva M, Siebert S (2005) Determination of Cr(VI) in Plants by Electrothermal Atomic Absorption Spectrometry after Leaching with Sodium Carbonate. Spectrochim Acta Part B Spectrosc 60(5):699–703. 10.1016/j.sab.2005.02.018

[6] Spanu D, Monticelli D, Binda G, Dossi C, Rampazzi L, Recchia S (2021) One-Minute Highly Selective Cr(VI) Determination at Ultra-Trace Levels: An ICP-MS Method Based on the on-Line Trapping of Cr(III). J Hazard Mater 412(15):125280. 10.1016/j.jhazmat.2021.125280 33550126 10.1016/j.jhazmat.2021.125280

[7] Liu Y, Liang P, Guo L (2005) Nanometer Titanium Dioxide Immobilized on Silica Gel as Sorbent for Preconcentration of Metal Ions Prior to Their Determination by Inductively Coupled Plasma Atomic Emission Spectrometry. Talanta 68(1):25–30. 10.1016/j.talanta.2005.04.035 18970280 10.1016/j.talanta.2005.04.035

[8] Shifera GA, Beyene TT, Gure A (2025] Effervescent-Assisted Deep Eutectic Solvent-Based Dispersive Liquid-Liquid Microextraction of Cr(VI) In Aqueous Samples. Bull Chem Soc Ethiop 39 (7): 1245–1256. 10.4314/bcse.v39i7.2

[9] Babazadeh S, Bisauriya R, Carbone M, Roselli L, Cecchetti D, Bauer EM, Sennato S, Prosposito P, Pizzoferrato R (2021) Colorimetric Detection of Chromium(VI) Ions in Water Using Unfolded-Fullerene Carbon Nanoparticles. Sensors 21(19):6353. 10.3390/s21196353 34640679 10.3390/s21196353 PMC8512488

[10] Goswami J, Saikia L, Hazarika P (2022) Carbon Dots-Decorated g-C_3_N_4_ as Peroxidase Nanozyme for Colorimetric Detection of Cr(VI) in Aqueous Medium. Chem Select 7(31):1–10. 10.1002/slct.202201963

[11] Adurty S, Sabbu JR (2015) Novel Catalytic Fluorescence Method for Speciative Determination of Chromium in Environmental Samples. J Anal Sci Technol 6(7):1–8. 10.1186/s40543-015-0044-7

[12] Zhang L, Xu C, Li B (2009) Simple and Sensitive Detection Method for Chromium(VI) in Water Using Glutathione-Capped CdTe Quantum Dots as Fluorescent Probes. Microchim Acta 166:61–68. 10.1007/s00604-009-0164-0

[13] Fang LY, Zheng JT, Chen ZZ (2016) Carbon Dot as Fluorescent Probe for Detection of Chromium(VI). Adv Eng Res 115:226–233. 10.2991/eesed-16.2017.29

[14] Patil AB, Chaudhary PL, Adhyapak PV (2024) Carbon Dots-Cadmium Sulfide Quantum Dots Nanocomposite for ‘on-off’ Fluorescence Sensing of Chromium(VI) Ions. RSC Adv 14(1):12923–12934. 10.1039/d4ra00436a 38650690 10.1039/d4ra00436a PMC11033546

[15] Yang WM, Liu F, Jin YT, Dong ZM, Zhao GC (2022) Efficient Reduction of Cr(VI) with Carbon Quantum Dots. ACS Omega 7(2):23555–23565. 10.1021/acsomega.2c02063 35847330 10.1021/acsomega.2c02063 PMC9280965

[16] Berhanu S, Habtamu F, Tadesse Y, Gonfa F, Tadesse T (2022) Fluorescence Sensor Based on Polyaniline Supported Ag-ZnO Nanocomposite for Malathion Detection. J Sens. 10.1155/2022/9881935. 1.–11

[17] Srithar A, Kannan JC, Senthil TS (2017) Preparation and Characterization of Ag Doped ZnO Nanoparticles and Its Antibacterial Applications Academic Discipline and Sub-Disciplines Nano Technology Type (Method/Approach). JAC 13:6273–6279

[18] Wang X, Li J (2021) Sol-Gel Fabrication of Ag-Coated ZnO Quantum Dots Nanocomposites with Excellent Photocatalytic Activity. Opt Mater 118:111235. 10.1016/j.optmat.2021.111235

[19] Geng S, Lin SM, Li NB, Luo HQ (2017) Polyethylene Glycol Capped ZnO Quantum Dots as a Fluorescent Probe for Determining Copper(II) Ion. Sens Actuator B-Chem 253:137–143. 10.1016/j.snb.2017.06.118

[20] Zou T, Xing X, Yang Y, Wang Z, Wang Z, Zhao R, Zhang X, Wang Y (2020) Water-Soluble ZnO Quantum Dots Modified by (3-Aminopropyl)Triethoxysilane: The Promising Fluorescent Probe for the Selective Detection of Cu^2+^ Ion in Drinking Water. J Alloys Compd 825(15):153904. 10.1016/j.jallcom.2020.153904

[21] Kaviya S, Kabila S, Jayasree KV (2017) Room Temperature Biosynthesis of Greatly Stable Fluorescent ZnO Quantum Dots for the Selective Detection of Cr^3+^ Ions. Mater Res Bull 95:163–168. 10.1016/j.materresbull.2017.07.025

[22] Liu X, Yang Y, Xing X, Wang Y (2018) Grey Level Replaces Fluorescent Intensity: Fluorescent Paper Sensor Based on ZnO Nanoparticles for Quantitative Detection of Cu^2+^ without Photoluminescence Spectrometer. Sens Actuator B-Chem 255(2):2356–2366. 10.1016/j.snb.2017.09.044

[23] Tong Z, Xing X, Yang Y, Hong P, Wang Z, Zhao R, Zhang X, Peng S, Wang Y (2019) Fluorescent ZnO Quantum Dots Synthesized with Urea for the Selective Detection of Cr^6+^ Ion in Water with a Wide Range of Concentrations. Methods Appl Fluoresc 7(3):035007. 10.1088/2050-6120/ab29c8 31195378 10.1088/2050-6120/ab29c8

[24] Daniel SCGK, Kumar A, Sivasakthi K, Thakur CS (2019) Handheld, Low-Cost Electronic Device for Rapid, Real-Time Fluorescence-Based Detection of Hg^2+^, Using Aptamer-Templated ZnO Quantum Dots. Sens Actuators B-Chem 290:73–78. 10.1016/j.snb.2019.03.113

[25] Ng SM, Wong DSN, Phung JHC, Chua HS (2013) Integrated Miniature Fluorescent Probe to Leverage the Sensing Potential of ZnO Quantum Dots for the Detection of Copper (II) Ions. Talanta 116:514–519. 10.1016/j.talanta.2013.07.031 24148438 10.1016/j.talanta.2013.07.031

[26] Thakur D, Sharma A, Rana DS, Thakur N, Singh D, Tamulevicius T, Andrulevicius M (2020) Facile Synthesis of Silver-Doped Zinc Oxide Nanostructures as Efficient Scaffolds for Detection of p-Nitrophenol. Chemosensors 8:108. 10.3390/chemosensors8040108

[27] Lamba R, Bhanjana G, Dilbaghi N, Gupta V, Kumar S (2025) Quantification of Lead through Rod-Shaped Silver-Doped Zinc Oxide Nanoparticles Using an Electrochemical Approach. Beilstein J Nanotechnol 16:422–434. 10.3762/BJNANO.16.33. 40166480 10.3762/bjnano.16.33 PMC11956069

[28] Saquib AM, Ahmad R, Khan MY, Ahmad A, Alshammari MB, Lee B (2025) Fabrication of an Ag-Doped ZnO Nanoparticle-Based Electrochemical Sensor for Arsenic Detection in Water. ECS J Solid State Sci Technol 14:077003. 10.1149/2162-8777/adeae1

[29] Hussain A, Fiaz S, Almohammedi A, Waqar A (2024) Optimizing Photocatalytic Performance with Ag-Doped ZnO Nanoparticles: Synthesis and Characterization. Heliyon 10:e35725. 10.1016/j.heliyon.2024.e35725 39170244 10.1016/j.heliyon.2024.e35725 PMC11336865

[30] Teshome T, Addisu KS, Gure A, Gonfa G (2024) Electrochemical Detection of Tryptophan in Fish and Pharmaceutical Supplement at Glassy Carbon Electrode Modified with Fe-Doped ZnO Nanoparticle. Electroanalysis 36(2):1–11. 10.1002/elan.202300237

[31] Ali IO, Nady H, Mohamed MI, Salama TM (2024) Fabrication and Characterization of ZnO and Ag/ZnO Nanoparticles for Efficient Degradation of Crystal Violet Dye in Aqueous Solution. J Indian Chem Soc 101(12):101480. 10.1016/j.jics.2024.101480

[32] Zhu X, Wang J, Yang D, Liu J, He L, Tang M, Feng W, Wu X (2021) Fabrication, Characterization and High Photocatalytic Activity of Ag-ZnO Heterojunctions under UV-Visible Light. RSC Adv 11:27257–27266. 10.1039/d1ra05060e 35480683 10.1039/d1ra05060e PMC9037622

[33] Bhosale A, Kadam J, Gade T, Sonawane K (2023) Efficient Photodegradation of Methyl Orange and Bactericidal Activity of Ag Doped ZnO Nanoparticles. J Indian Chem Soc 100(2):100920. 10.1016/j.jics.2023.100920

[34] Ahmad I, Ahmed E, Ahmad M (2019) The Excellent Photocatalytic Performances of Silver Doped ZnO Nanoparticles for Hydrogen Evolution. SN Appl Sci 1(327):327. 10.1007/s42452-019-0331-9

[35] Ramasamy B, Jeyadharmarajan J, Chinnaiyan P (2021) Novel Organic Assisted Ag-ZnO Photocatalyst for Atenolol and Acetaminophen Photocatalytic Degradation under Visible Radiation: Performance and Reaction Mechanism. Environ Sci Pollut Res Int 28:39637–39647. 10.1007/s11356-021-13532-2 33763832 10.1007/s11356-021-13532-2 PMC7990384

[36] Kadam AN, Bhopate DP, Kondalkar VV, Majhi SM, Bathula CD, Tran AV, Lee SW (2018) Facile Synthesis of Ag-ZnO Core–Shell Nanostructures with Enhanced Photocatalytic Activity. J Ind Eng Chem 61:78–86. 10.1016/j.jiec.2017.12.003

[37] Kaur A, Ibhadon AO, Kansal SK (2017) Photocatalytic Degradation of Ketorolac Tromethamine (KTC) Using Ag-Doped ZnO Microplates. J Mater Sci 52:5256–5267. 10.1007/s10853-017-0766-6

[38] Rathika A, Irine TM (2022) Synthesis Of Silver (Ag) Doped Zinc Oxide (ZnO) Nanoparticles As Efficient Photocatalytic Activity For Degradation Methylene Blue Dye. J Adv Res 13(02):129–135. 10.55218/JASR.202213217

[39] Kareem MA, Bello IT, Shittu HA, Sivaprakash P (2022) Synthesis, Characterization, and Photocatalytic Application of Silver Doped Zinc Oxide Nanoparticles. Clean Mater 3:4–10. 10.1016/j.clema.2022.10004

[40] Hussain A, Fiaz S, Almohammedi A, Waqar A (2024) Optimizing Photocatalytic Performance with Ag -Doped ZnO Nanoparticles: Synthesis and Characterization. Heliyon 10(15):e35725. 10.1016/j.heliyon.2024.e35725 39170244 10.1016/j.heliyon.2024.e35725 PMC11336865

[41] Gao D, Zhang A, Lyu B, Ma J (2024) Visual and Rapid Fluorescence Sensing for Hexavalent Chromium by Hydroxypropyl Chitosan Passivated Bismuth – Based Perovskite Quantum Dots. Microchim Acta 191:219. 10.1007/s00604-024-06251-1 10.1007/s00604-024-06251-1 38530477

[42] Li Q, Yang D, Yang Y (2021) Spectrofluorimetric Determination of Cr(VI) and Cr(III) by Quenching Effect of Cr(III) Based on the Cu-CDs with Peroxidase-Mimicking Activity. Spectrochim Acta Mol Biomol Spectrosc 244:118882. 10.1016/j.saa.2020.118882 10.1016/j.saa.2020.118882 32919158

[43] Nelson DJ, Gowthaman NSK, Sinduja B, Sethuraman MG (2025) A Facile and Rapid Detection of Cr (VI) and Ascorbic Acid Using N, S-Co-Functionalized Carbon Dots : A Dual-Mode ’ on-off-on ’ Fluorescent Sensor. Talanta Open 11:100472. 10.1016/j.talo.2025.100472

[44] Sheng L, Huangfu B, Xu Q, Tian W, Li Z, Meng A, Tan S (2019) A Highly Selective and Sensitive Fl Uorescent Probe for Detecting Cr (VI) and Cell Imaging Based on Nitrogen-Doped Graphene Quantum Dots. J Alloys Compd 820:153191. 10.1016/j.jallcom.2019.153191

[45] Liu X, Li T, Wu Q, Yan X, Wu C, Chen X, Zhang G (2017) Carbon Nanodots as a Fl Uorescence Sensor for Rapid and Sensitive Detection of Cr (VI) and Their Multifunctional Applications. Talanta 165:216–222. 10.1016/j.talanta.2016.12.037 28153245 10.1016/j.talanta.2016.12.037

[46] Feng S, Gao Z, Liu H, Huang J, Li X, Yang Y (2019) Feasibility of Detection Valence Speciation of Cr(III) and Cr(VI) in Environmental Samples by Spectrofluorimetric Method with Fluorescent Carbon Quantum Dots. Spectrochim Acta Mol Biomol Spectrosc 212:286–292. 10.1016/j.saa.2018.12.055 10.1016/j.saa.2018.12.055 30660836

[47] Ebrahim S, Shokry A, Khalil MMA, Ibrahim H, Soliman M (2020) Polyaniline/AgNanoparticles/Graphene Oxide Nanocomposite Fluorescent Sensor for Recognition of Chromium(VI) Ions. Sci Rep 10:13617. 10.1038/s41598020-70678-8 10.1038/s41598-020-70678-8 PMC7423961 32788693

[48] Tian YL, Ji YY, Zou X, Chen QM, Zhang SL, Gong ZJ (2022) N, P Co – Doped Carbon Dots as Multifunctional Fluorescence Nano – Sensor for Sensitive and Selective Detection of Cr (VI) and Ascorbic Acid. J Anal Test 6:335–345. 10.1007/s41664-022-00213-3

[49] Sudan S, Kaushal J, Singh TG, Mahmoud MH, Alexiou A, Papadakis M, Fetoh MEAE (2025) Eco – Friendly Sensing of Hexavalent Chromium Ions via Copper – Doped Carbon Quantum Dots: A Fluorescent Probe for Water. Saf Microchimica Acta 192:88. 10.1007/s00604-024-06939-4 10.1007/s00604-024-06939-4 PMC11735500 39815044

[50] Zhang Z, Fan Z (2021) Molecular and Biomolecular Spectroscopy Morphological Analysis of Chromium in Carbon Quantum Dots Pairs Co-Doped with Zirconium and Nitrogen and Their Applications in Imaging of Living Cells. Spectrochim Acta Mol Biomol Spectrosc 250:119248. 10.1016/j.saa.2020.119248 10.1016/j.saa.2020.119248 33288432

[51] Ashebir ME, Tesfamariam GM, Nigussie GY, Gebreab TW (2018) Structural, Optical, and Photocatalytic Activities of Ag-Doped and Mn‐Doped ZnO Nanoparticles. J Nanomater 9425938. 10.1155/2018/9425938

